# Unravelling the influence of affective stimulation on functional neurological symptoms: a pilot experiment examining potential mechanisms

**DOI:** 10.1136/jnnp-2023-332364

**Published:** 2023-11-14

**Authors:** Susannah Pick, LS Merritt Millman, Emily Ward, Eleanor Short, Biba Stanton, AAT Simone Reinders, Joel S Winston, Timothy R Nicholson, Mark J Edwards, Laura H Goldstein, Anthony S David, Trudie Chalder, Matthew Hotopf, Mitul A Mehta

**Affiliations:** 1 King's College London, Institute of Psychiatry, Psychology and Neuroscience, London, UK; 2 King's College Hospital NHS Foundation Trust, London, UK; 3 Institute of Mental Health, University College London, London, UK; 4 South London and Maudsley NHS Foundation Trust, London, UK

**Keywords:** FUNCTIONAL NEUROLOGICAL DISORDER, PSYCHOLOGY, EXPERIMENTAL, CONVERSION DISORDER, AUTONOMIC, NEUROPSYCHIATRY

## Abstract

**Background:**

Differences in affective processing have previously been shown in functional neurological disorder (FND); however, the mechanistic relevance is uncertain. We tested the hypotheses that highly arousing affective stimulation would result in elevated subjective functional neurological symptoms (FNS), and this would be associated with elevated autonomic reactivity. The possible influence of cognitive detachment was also explored.

**Method:**

Individuals diagnosed with FND (motor symptoms/seizures; n=14) and healthy controls (n=14) viewed Positive, Negative and Neutral images in blocks, while passively observing the stimuli (‘Watch’) or detaching themselves (‘Distance’). The FND group rated their primary FNS, and all participants rated subjective physical (arousal, pain, fatigue) and psychological states (positive/negative affect, dissociation), immediately after each block. Skin conductance (SC) and heart rate (HR) were monitored continuously.

**Results:**

FNS ratings were higher after Negative compared with Positive and Neutral blocks in the FND group (p=0.002, η_p_^2^=0.386); however, this effect was diminished in the Distance condition relative to the Watch condition (p=0.018, η_p_^2^=0.267). SC and/or HR correlated with FNS ratings in the Negative-Watch and Neutral-Distance conditions (r values=0.527–0.672, p values=0.006–0.035). The groups did not differ in subjective affect or perceived arousal (p values=0.541–0.919, η_p_^2^=<0.001–0.015).

**Conclusions:**

Emotionally significant events may exert an influence on FNS which is related to autonomic activation rather than altered subjective affect or perceived arousal. This influence may be modulated by cognitive detachment. Further work is needed to determine the relevance and neural bases of these processes in specific FND phenotypes.

**WHAT IS ALREADY KNOWN ON THIS TOPIC**

- Functional neurological disorder (FND) samples show differences in affective responsivity and awareness; however, the direct influence of affective events on functional neurological symptoms (FNS) has not previously been demonstrated.

**WHAT THIS STUDY ADDS**

- We piloted an experimental task allowing us to provide the first evidence of a direct influence of negative affective stimulation on momentary subjective FNS, which was associated with autonomic activation rather than changes in subjective affect or perceived arousal.

**HOW THIS STUDY MIGHT AFFECT RESEARCH, PRACTICE OR POLICY**

- Our findings support models proposing roles for affective/autonomic mechanisms in FND, indicating that interventions aimed at improving awareness, integration and regulation of autonomic signals might be beneficial for some individuals with the disorder.

## Background

The pathophysiological mechanisms contributing to the generation of functional neurological symptoms (FNS) have not yet been explicated completely, although current models suggest possible interacting roles for altered attention, predictive processing, sense of agency, executive functioning, interoception and emotional processing.^1^ Altered affective processing and/or autonomic arousal, specifically, have been recurrent themes in theoretical models of functional neurological disorder (FND).^2–6^

The relevance of affective processing and autonomic differences in FND has been broadly supported by empirical evidence. Many individuals with FND report emotionally salient experiences prior to the onset of the disorder or immediately preceding FNS occurrence/exacerbation.^7–10^ Experimental evidence has shown that some FND samples exhibit discrepant autonomic and/or subjective responses to affective images,^11 12^ reduced recognition and enhanced attentional biases for emotional facial expressions,^13–15^ and altered interoceptive accuracy, insight and/or sensibility.^16–18^ Distinct patterns of neural activation have been observed during affective processing and psychosocial stress induction in FND samples^19–21^, in addition to disturbances in resting-state limbic, autonomic and hypothalamic-pituitary-adrenal axis functioning in adult and paediatric FND samples.^13 15 22–26^ Peri-ictal objective and/or subjective markers of autonomic activation have also been reported in individuals with functional seizures (FS).^10 27–29^ Nevertheless, findings have been variable and significant methodological limitations identified in this literature.^4 30^ Notably, few studies have examined whether affective processing differences or autonomic arousal have a direct influence on sensorimotor function or FNS.^31 32^

Further research is needed to unravel possible interactions between affective processing, autonomic arousal and FNS, with close attention to their temporal relationships. Examining these interactions will help determine whether affective processing differences and autonomic arousal exert a causal influence on FNS, rather than simply representing correlates of the disorder.

### Aims and hypotheses

We aimed to assess the feasibility and validity of an experimental task designed to examine the influence of affective stimulation and autonomic arousal on FNS severity.

We tested the following hypotheses^4^:

1. Individuals with FND would exhibit elevated autonomic arousal (skin conductance (SC)/heart rate (HR)) versus healthy controls (HCs) during affective stimulation (positive/negative).
2. The FND group would report increased FNS severity immediately following affective stimulation, relative to a neutral control condition.
3. Autonomic arousal during affective stimulation would be associated with FNS severity ratings.
4. The relationship between autonomic arousal and subjective affect/perceived arousal would be weaker in the FND group than HCs.

There were also some exploratory aspects to this study. Dissociative tendencies are elevated in FND^33^ and dissociation may contribute to the generation of FNS^17 34^; therefore, we attempted to experimentally model dissociative states within the task. Pain and fatigue are common complaints in FND^35 36^ and may share common underlying mechanisms.^37^ We included momentary probes to assess dissociation, pain and fatigue within the task, to examine the influence of affective stimulation on these other common symptoms.

## Methods

This experiment was part of a larger pilot study investigating aetiological factors and mechanisms in individuals with FND with motor symptoms and seizures. Data were collected from July to October 2022.

### Participants

In total, 14 participants diagnosed with FND with motor symptoms (FMS, n=11) or seizures (FS, n=3) as their primary FNS were compared with HCs (n=14). This sample size was considered adequate to evaluate the feasibility of the paradigm and approximate effect sizes.

The recruitment/screening processes and eligibility criteria for the study are detailed in online supplemental table 1 and previous publications.^16 38^ The FND sample were recruited through FND charities (FND Hope UK, FND Action) and social media (eg, Twitter). HCs were recruited from the local area. Background screening was conducted remotely (SP), involving a detailed psychosocial/medical history interview and an abbreviated SCID-5-RV.^39^ Participants in the FND group provided medical documentation to confirm a primary diagnosis of FMS/FS. Diagnostic and Statistical Manual of Mental Disorders (fifth edition)^40^ criteria for FND were also confirmed during the screening interview.

10.1136/jnnp-2023-332364.supp1 Supplementary data

At baseline, participants with FMS were asked to specify their primary motor symptom and those with FS were asked to identify their most consistent seizure warning symptom, so that these symptoms could be reported throughout the task with momentary probes (see below). Individuals with FS without seizure warning symptoms were ineligible because it would not be possible to monitor variability in their FNS during the experiment. We described seizure warnings as any experience or symptom that typically occurs prior to the onset of a FS for that individual. This could include motor or other symptoms (eg, shaking, pain, intense fatigue), sensations (eg, a taste, smell), emotional states (eg, fear, anxiety) or other psychological phenomena (eg, a general feeling of going into a seizure-like state). Alterations in awareness, such as detachment, could also be classified as a seizure warning sign, but it was a prerequisite that participants did not lose awareness or the ability to respond completely, because they were required to report on their symptoms and stay alert throughout the task.

All participants were reimbursed with a £50 shopping voucher.

### Materials and measures

#### Self-report measures

Validated questionnaires (online supplemental table 2) assessed adverse life events,^41^ dissociative tendencies,^42^ anxiety,^43^ depression,^44^ alexithymia^45^ and physical symptom burden.^46^ A bespoke Functional Neurological Symptoms Questionnaire (online supplemental table 3) captured the range, severity and impact of FNS experienced in the FND sample.

#### Affective images task

The experiment had a mixed between-groups and within-groups design. The within-groups factors were image-type (Positive/Negative/Neutral) and task-instruction (Watch/Distance). Participants were asked to either passively observe the images (Watch) or voluntarily detach themselves (Distance) (online supplemental table 4). Affective images were selected from the International Affective Picture System^47^ based on normative valence and arousal ratings (online supplemental table 5).

The experiment was administered using E-Prime V.3.0 (https://pstnet.com/products/e-prime/), consisting of 12 blocks of 10 images. We adopted a block design to induce longer-term changes in emotional state and autonomic arousal than event-related designs.^48^ Two blocks each of the following conditions were administered: Negative-Watch; Negative-Distance; Positive-Watch; Positive-Distance; Neutral-Watch; Neutral-Distance. The order of blocks was pseudorandomised.

Each block commenced with the task-instruction (Watch/Distance) presented for 2000 ms. Ten images of the same type (Positive/Negative/Neutral) were then presented in a random order (6000 ms each), all preceded by a fixation cross (500 ms) and the word ‘Watch’ or ‘Distance’ (1000 ms). Interstimulus intervals were jittered (1250–2000 ms).

Participants completed momentary subjective assessments (table 1) immediately after each block, followed by an interblock interval (25–35 s) during which the instruction ‘rest’ appeared. Momentary FNS severity (FND group only), pain, fatigue and arousal were assessed with items developed by the research team and our FND Patient and Carer Advisory Panel. Items adapted from the Positive and Negative Affect Schedule^49^ measured momentary affect, and items modified from the Clinician Administered Dissociative States Scale^50^ assessed dissociative states. The order of the momentary probes was randomised, aside for the FNS ratings which always came first. Participants responded manually using a Likert scale from 1 (not at all) to 7 (extremely).

**Table 1:** Subjective momentary assessments

Dependent variable (all rated 1–7 Likert)	Item wording: Right now, at the present moment….
Functional motor symptoms	I am experiencing my primary FND symptom.
Functional seizures	I am experiencing my primary seizure warning symptom.
Dissociation-depersonalisation	I feel disconnected from my own body. I feel separated from what is happening to me, like an actor in a movie, or a robot.
Dissociation-derealisation	Things seem unreal to me, as if I am in a dream. It seems like I am looking at the world through a fog.
Dissociation-amnesia	I cannot account for things that have recently happened. I feel spaced out, and/or have lost track of what is going on.
Affect-positive	I feel… Enthusiastic Determined Excited Alert Proud Strong
Affect-negative	I feel… Scared Upset Nervous Ashamed Irritable Hostile
Arousal	I feel bodily arousal.*
Pain	I am in bodily pain.
Fatigue	I feel tired.

*Instructions were given to participants to ensure that they understood the meaning of bodily arousal (ie, sympathetic nervous system activation), with examples (eg, dry mouth, racing heart, sweat response).

FND, functional neurological disorder.

#### Psychophysiological measures

Psychophysiological measures were recorded using a Powerlab data acquisition system, with LabChart V.8 software (https://www.adinstruments.com/). Recordings were acquired throughout the baseline period and experimental task, sampled at 1 KHz.

##### Skin conductance (SC)

SC was measured with 8 mm Ag/AgCl electrodes, filled with electrode paste and applied to the distal phalanges of the index and middle digits of the non-dominant hand.^51^ SC was calibrated to measure a range of 0–50 microSiemens for each participant prior to the baseline recording.

##### Heart rate (HR)

After skin preparation, electrocardiography electrodes were placed in an Einthoven triangle (left arm/right arm/left leg). A range of 1–2 mV was adopted and adjusted to individual participants if necessary. HR in beats per minute was computed from interbeat intervals.

### Procedure

Following a screening interview and online questionnaire pack described in detail elsewhere,^16 38^ participants attended a laboratory testing session. All participants completed this experiment between 14:00 and 16:00 in the same testing room, following approximately 1–2 hours of other cognitive/experimental tasks.

The psychophysiology electrodes were first attached and participants were seated for 3–5 min, before a 5 min baseline recording. Participants then completed baseline subjective momentary assessments (table 1) and were presented with written task instructions onscreen, followed by six practice images. The experimenter (SP) answered questions, checked participants’ understanding of the task and remained present throughout the procedures.

### Data processing and analysis

Data analyses were conducted in SPSS (V.29, IBM) by SP and verified independently by LSMM. Values of 2.5 SD above/below the group mean for each variable were considered outliers and winsorized. Hypothesis-driven tests were one tailed (alpha p≤0.05) and exploratory tests were two tailed (alpha p≤0.01). Effect sizes were Hedge’s g, r or partial η^2^.

Sociodemographic and clinical variables were analysed with between-group tests, including t-tests, Mann-Whitney or chi-squared tests, as appropriate.

Momentary assessment scores were averaged across the two blocks for each condition. A two-way repeated-measures analysis of variance (ANOVA) assessed the influence of image-type (Positive/Negative/Neutral) and task-instruction (Watch/Distance) on subjective FNS ratings (FND group only). Three-way mixed ANOVAs were conducted for all other momentary subjective variables, with group as the between-group factor (FND/HC), and task-instruction (Watch/Distance) and image-type (Positive/Negative/Neutral) as within-group variables. Post hoc t-tests adopted Bonferroni corrections.

SC and HR data were screened visually for artefacts and segments of contaminated data were excluded prior to analysis. SC and HR data from the periods when the subjective probes were completed were excluded from analysis due to probable movement artefact introduced by the manual responses. SC and HR data for one participant from each group were excluded due to inadequate data quality. Baseline SC/HR scores were calculated from the mean values obtained during the last 2 min of the baseline recording. Mean SC/HR scores were calculated for each picture-viewing block by subtracting baseline means from block means. Scores were averaged across the two blocks for each condition, and analysed with three-way mixed ANOVAs (described above).

To examine the hypothesised relationship between momentary FNS severity and elevated autonomic arousal, correlations were computed between momentary FNS ratings and SC/HR for the conditions in which the highest FNS ratings were observed. Correlations were also carried out to test the hypothesis that the relationship between SC/HR and momentary subjective affect and arousal would be diminished in the FNS group relative to HCs.

Exploratory correlational analyses assessed possible relationships between key experimental dependent variables (momentary FNS ratings, SC/HR) and sociodemographic/clinical variables that differed significantly between groups. Pearson’s or Spearman’s coefficients were computed as appropriate.

## Results

### Sample characteristics

All participants in the FND group reported experiencing FMS or FS as their primary symptom, but they also reported at least one additional FNS (table 2).

**Table 2:** Demographic and clinical characteristics

	FND (n=14)	HC (n=14)	Test statistics (df)	p value	Effect size
Age: M (SD)	39.2 (9.3)	39.8 (11.1)	t(26)=−0.148	0.883	g=0.054
Gender (female): n (%)	10 (71)	11 (79)		1.000*	
Handedness (right): n (%)	12 (86)	13 (93)		1.000*	
Body mass index	28.0 (8.1)	26.3 (5.5)	t(26)=0.646	0.524	g=0.237
Relationship status (married/cohabiting): n (%)	9 (64)	6 (43)		0.450*	
Education (post compulsory): n (%)	13 (93)	14 (100)		1.000*	
Ethnicity (white): n (%)	12 (86)	9 (64)		0.390*	
Employment status (employed/full-time education): n (%)	4 (29)	13 (93)		0.001*	
Full-scale IQ score: M (SD)	102.1 (14.6)	105.6 (9.6)	t(26)=−0.764	0.452	g=0.280
Primary FNS: n (%)	FMS=11 (79) FS=3 (21)				
Additional FNS:†n (%)	Sensory=14 (100) Dizziness=12 (86) Speech/swallowing=9 (65) Cognitive=11 (79)				
FNSQ					
Severity (1–7): M (SD)	3.9 (1.0)				
Impact (1–7): M (SD)	3.9 (0.8)				
Current mental health diagnosis: n (%)	8 (57)	1 (7)		0.013*	
Current physical health diagnosis (not FND): n (%)	10 (71)	4 (29)		0.057*	
Medication: n (%)	13 (93)	4 (29)		0.001*	

*Fisher’s exact.

†All participants in the FNS group reported>1 FNS.

df, degrees of freedom; FMS, functional motor symptoms; FND, functional neurological disorder; FNS, functional neurological symptoms; FNSQ, Functional Neurological Symptoms Questionnaire; FS, functional seizures; HC, healthy controls; IQ, intelligence quotient; M, mean; SD, standard deviation.

The groups did not differ significantly on most possible confounding variables; however, a significantly greater proportion of the FND group reported mental health diagnoses and taking medication, and fewer of the FND group were in employment/full-time education. This FND sample reported significantly greater depression, anxiety, somatoform dissociation, depersonalisation, alexithymia and physical symptom burden, compared with HCs (online supplemental table 2).

### Subjective momentary assessments

#### Physical states

##### Functional neurological symptoms

The average momentary FNS severity rating at baseline was in the mild–moderate range. The task-based ANOVA yielded a significant main effect of image-type. FNS ratings were significantly higher following Negative compared with Positive (MD=0.55, p=0.009) and Neutral blocks (MD=0.38, p=0.048). However, FNS ratings did not differ for Positive and Neutral blocks (MD=0.18, p=0.599) (table 3).

**Table 3:** Subjective momentary assessment statistics—physical states

	FND (n=14)	HC (n=14)	Test/effect	Test statistics (df)	p value	Effect size
**FNS severity**						
Baseline: M (SD)	3.36 (1.15)					
Task: M (SD)			**ANOVA**			
Positive-Watch	2.82 (1.41)		Image-type	F(2, 26)=8.17	**0.002**	η_p_^2^=0.386
Positive-Distance	3.00 (1.58)		Task-instruction	F(1, 13)=1.24	0.286	η_p_^2^=0.087
Negative-Watch	3.64 (1.69)		Image-type × task-instruction	F(2, 26)=4.74	**0.018**	η_p_^2^=0.267
Negative-Distance	3.29 (1.50)					
Neutral-Watch	2.82 (1.30)					
Neutral-Distance	3.36 (1.60)					
**Pain**			**Mann-Whitney**			
Baseline: Mdn (IQR)	3.00 (3.00)	1.00 (0.00)	Group	U=16.5, z=−4.09	**<0.001**	r=0.773
Task: M (SD)			**ANOVA**			
Positive-Watch	3.00 (1.58)	1.21 (0.43)	Group	F(1, 26)=20.90	**<0.001**	η_p_^2^=0.446
Positive-Distance	3.29 (1.79)	1.14 (0.31)	Image-type	F(2, 52)=3.45	**0.039**	η_p_^2^=0.117
Negative-Watch	3.43 (1.69)	1.21 (0.38)	Task-instruction	F(1, 26)=1.42	0.244	η_p_^2^=0.052
Negative-Distance	3.46 (1.81)	1.18 (0.37)	Image-type × group	F(2, 52)=2.32	0.109	η_p_^2^=0.082
Neutral-Watch	3.14 (1.74)	1.14 (0.36)	Image-type × task-instruction	F(2, 52)=0.51	0.604	η_p_^2^=0.019
Neutral-Distance	3.29 (1.73)	1.14 (0.36)	Group × task-instruction	F(1, 26)=3.64	0.067	η_p_^2^=0.123
			Image-type × task-instruction × group	F(2, 52)=0.94	0.396	η_p_^2^=0.035
**Fatigue**			**Mann-Whitney**			
Baseline: Mdn (IQR)	5.0 (3.25)	2.0 (0.25)	Group	U=33, z=−3.13	**0.002**	r=0.592
Task: M (SD)			**ANOVA**			
Positive-Watch	4.18 (1.62)	2.36 (1.20)	Group	F(1, 26)=11.70	**0.002**	η_p_^2^=0.310
Positive-Distance	4.61 (1.46)	2.75 (1.40)	Image-type	F(2, 52)=1.51	0.230	η_p_^2^=0.055
Negative-Watch	4.5 (1.41)	2.86 (1.35)	Task-instruction	F(1, 26)=13.80	**<0.001**	η_p_^2^=0.347
Negative-Distance	4.64 (1.60)	2.89 (1.46)	Image-type × group	F(2, 52)=0.37	0.692	η_p_^2^=0.014
Neutral-Watch	4.14 (1.55)	2.36 (1.08)	Image-type × task-instruction	F(2, 52)=5.62	**0.006**	η_p_^2^=0.178
Neutral-Distance	4.71 (1.58)	3.32 (1.71)	Group × task-instruction	F(1, 26)=0.13	0.717	η_p_^2^=0.005
			Image-type × task-instruction × group	F(2, 52)=0.89	0.416	η_p_^2^=0.033
**Arousal**			**Mann-Whitney**			
Baseline: Mdn (IQR)	1.5 (1.25)	1.0 (1.00)	Group	U=71, z=−1.43	0.153	r=0.270
Task: M (SD)			**ANOVA**			
Positive-Watch	1.93 (0.87)	1.96 (0.97)	Group	F(1, 26)=0.07	0.797	η_p_^2^=0.003
Positive-Distance	1.86 (0.82)	1.71 (0.83)	Image-type*	F(1.76, 45.7)=5.04	**0.013**	η_p_^2^=0.162
Negative-Watch	1.75 (0.89)	1.61 (0.94)	Task-instruction	F(1, 26)=0.71	0.408	η_p_^2^=0.026
Negative-Distance	1.54 (0.75)	1.71 (0.99)	Image-type × group*	F(1.76, 45.7)=0.26	0.742	η_p_^2^=0.010
Neutral-Watch	1.57 (0.65)	1.36 (0.66)	Image-type × task-instruction*	F(1.78, 46.3)=0.69	0.493	η_p_^2^=0.026
Neutral-Distance	1.54 (0.72)	1.43 (0.73)	Group × task-instruction	F(1, 26)=0.29	0.597	η_p_^2^=0.011
			Image-type × task-instruction × group*	F(1.78, 46.3)=1.33	0.272	η_p_^2^=0.049

*Huynh-Feldt correction for non-sphericity.

ANOVA, analysis of variance; df, degrees of freedom; FND, functional neurological disorder; HC, healthy controls; IQR, interquartile range; M, mean; Mdn, median; SD, standard deviation; η_p_^2^, partial eta squared.

There was a significant image-type × task-instruction interaction. FNS ratings were significantly elevated after Negative compared with both Positive (MD=0.82, p=0.035) and Neutral blocks (MD=0.82, p=0.032) in the Watch condition. In the Distance condition, FNS ratings were only higher for Negative compared with Positive (MD=0.29, p=0.017), but not Neutral blocks (MD=0.07, p=1.000). Mean ratings indicated that FNS were elevated after the Neutral-Distance blocks to a similar degree as the Negative-Distance blocks.

##### Pain

Pain ratings were significantly higher in the FND group compared with HCs at baseline and during the task. The task-based main effect of image-type was also significant. Across the sample, pain ratings were higher following Negative (M=2.32, SE=0.236) compared with Positive (M=2.16, SE=0.228) or Neutral blocks (M=2.18, SE=0.232), although these differences were not significant following Bonferroni correction.

##### Fatigue

The FND group reported significantly greater fatigue than HCs at baseline and during the task. There was a significant main effect of task-instruction, revealing significantly higher fatigue ratings for the Distance condition than the Watch condition, across the whole sample.

The interaction between image-type and task-instruction was also significant, across the complete sample. Fatigue ratings were higher for Negative (M=3.67, SE=0.261) compared with Positive (M=3.27, SE=0.270) and Neutral blocks (M=3.25, SE=0.253) in the Watch condition. However, in the Distance condition, fatigue ratings were high across image-types and did not differ significantly (all scores > 3.68).

##### Subjective arousal

The FND and HC groups reported comparable physiological arousal at baseline and during the task. There was a significant main effect of image-type on arousal ratings across the sample, with ratings significantly higher following Positive compared with Neutral blocks (MD=0.39, p=0.020), but not compared with Negative blocks (MD=0.21, p=0.440).

#### Psychological states

##### Affect

Positive affect ratings did not differ between groups at baseline or during the task. However, the main effects of image-type and task-instruction were significant, across the entire sample. The main effect of image-type was due to elevated positive affect ratings following Positive compared with Negative (MD=0.37, p=0.003) and Neutral blocks (MD=0.24, p=0.035); positive affect ratings did not differ between Negative and Neutral blocks (MD=0.13, p=0.608). The main effect of task-instruction was due to significantly elevated positive affect ratings following the Watch condition compared with the Distance condition (MD=0.22, p<0.001) (table 4).

**Table 4:** Subjective momentary assessment statistics—psychological states

	FND (n=14)	HC (n=14)	Test/effect	Test statistics (df)	P value	Effect size
**Positive affect**			**Independent samples t-test**			
Baseline: M (SD)	3.42 (1.17)	4.17 (1.06)	Group	t(26)=−1.77	0.088	g=0.650
Task: M (SD)			**ANOVA**			
Positive-Watch	3.58 (1.28)	3.48 (1.21)	Group	F(1, 26)=0.01	0.919	η_p_^2^=0.000
Positive-Distance	3.29 (1.23)	3.22 (1.27)	Image-type	F(2, 52)=7.66	**0.001**	η_p_^2^=0.228
Negative-Watch	3.07 (1.29)	3.05 (1.37)	Task-instruction	F(1, 26)=14.67	**<0.001**	η_p_^2^=0.361
Negative-Distance	2.91 (1.31)	3.08 (1.35)	Image-type × task-instruction	F(2, 52)=3.09	0.054	η_p_^2^=0.106
Neutral-Watch	3.39 (1.29)	3.23 (1.52)	Group × task-instruction	F(1, 26)=0.54	0.471	η_p_^2^=0.020
Neutral-Distance	3.06 (1.29)	2.94 (1.54)	Image-type × task-instruction × group	F(2, 52)=0.30	0.744	η_p_^2^=0.011
**Negative affect**			**Mann-Whitney**			
Baseline: Mdn (IQR)	1.17 (0.50)	1.0 (0.17)	Group	U=71, z=−1.33	0.185	r=0.251
Task: M (SD)			**ANOVA**			
Positive-Watch	1.32 (0.49)	1.10 (0.18)	Group	F(1, 26)=0.38	0.541	η_p_^2^=0.015
Positive-Distance	1.30 (0.27)	1.12 (0.16)	Image-type*	F(1.13, 29.4)=17.07	**<0.001**	η_p_^2^=0.396
Negative-Watch	1.69 (0.51)	1.90 (1.05)	Task-instruction	F(1, 26)=0.47	0.497	η_p_^2^=0.018
Negative-Distance	1.73 (0.66)	1.75 (0.94)	Image-type × group*	F(1.13, 29.4)=1.22	0.285	η_p_^2^=0.045
Neutral-Watch	1.18 (0.20)	1.05 (0.10)	Image-type × task-instruction†	F(1.56, 40.6)=2.46	0.11	η_p_^2^=0.086
Neutral-Distance	1.35 (0.37)	1.13 (0.25)	Group × task-instruction	F(1, 26)=1.44	0.241	η_p_^2^=0.052
			Image-type × task-instruction × group†	F(1.56, 40.6)=0.96	0.373	η_p_^2^=0.036
**Dissociation-derealisation**			**Mann-Whitney**			
Baseline: Mdn (IQR)	1.0 (2.25)	1.0 (0.00)	Group	U=65, z=−1.90	0.057	r=0.359
Task: M (SD)			**ANOVA**			
Positive-Watch	1.55 (1.07)	1.0 (0.00)	Group	F(1, 26)=4.20	0.051	η_p_^2^=0.139
Positive-Distance	1.82 (1.21)	1.13 (0.29)	Image-type†	F(1.62, 42.1)=1.33	0.271	η_p_^2^=0.049
Negative-Watch	1.77 (1.16)	1.14 (0.29)	Task-instruction	F(1, 26)=6.19	**0.020**	η_p_^2^=0.192
Negative-Distance	1.82 (1.21)	1.16 (0.32)	Image-type × group†	F(1.62, 42.1)=0.24	0.738	η_p_^2^=0.009
Neutral-Watch	1.54 (0.99)	1.0 (0.00)	Image-type × task-instruction†	F(1.82, 47.4)=3.23	0.053	η_p_^2^=0.110
Neutral-Distance	1.86 (1.28)	1.27 (0.53)	Group × task-instruction	F(1, 26)=0.30	0.588	η_p_^2^=0.011
			Image-type × task-instruction × group†	F(1.82, 47.4)=0.16	0.837	η_p_^2^=0.006
**Dissociation-depersonalisation**			**Mann-Whitney**			
Baseline: Mdn (IQR)	1.0 (0.63)	1.0 (0.63)	Group	U=94.5, z=−0.19	0.846	r=0.037
Task: M (SD)			**ANOVA**			
Positive-Watch	1.52 (0.78)	1.04 (0.09)	Group	F(1, 26)=2.86	0.103	η_p_^2^=0.099
Positive-Distance	1.57 (0.82)	1.29 (0.60)	Image-type*	F(1.24, 32.3)=2.65	0.107	η_p_^2^=0.092
Negative-Watch	1.68 (0.92)	1.30 (0.51)	Task-instruction	F(1, 26)=3.00	0.095	η_p_^2^=0.103
Negative-Distance	1.66 (0.86)	1.38 (0.90)	Image-type × group*	F(1.24, 32.3)=0.65	0.457	η_p_^2^=0.025
Neutral-Watch	1.46 (0.78)	1.07 (0.18)	Image-type × task-instruction†	F(1.63, 42.3)=3.37	0.053	η_p_^2^=0.115
Neutral-Distance	1.82 (0.89)	1.25 (0.55)	Group × task-instruction	F(1, 26)=0.04	0.837	η_p_^2^=0.002
			Image-type × task-instruction × group†	F(1.63, 42.3)=2.16	0.137	η_p_^2^=0.077
**Dissociation-amnesia**			**Mann-Whitney**			
Baseline: Mdn (IQR)	1.5 (0.63)	1.0 (0.13)	Group	U=51.5, z=−2.42	**0.016**	r=0.457
Task: M (SD)			**ANOVA**			
Positive-Watch	1.68 (0.93)	1.05 (0.11)	Group	F(1, 26)=6.87	**0.014**	η_p_^2^=0.209
Positive-Distance	1.71 (0.83)	1.13 (0.21)	Image-type*	F(1.15, 29.9)=1.97	0.170	η_p_^2^=0.071
Negative-Watch	1.73 (0.87)	1.16 (0.27)	Task-instruction	F(1, 26)=4.39	**0.046**	η_p_^2^=0.145
Negative-Distance	1.86 (1.04)	1.16 (0.32)	Image-type × group*	F(1.15, 29.9)=0.36	0.585	η_p_^2^=0.014
Neutral-Watch	1.63 (0.86)	1.05 (0.11)	Image-type × task-instruction*	F(1.7, 44.2)=3.79	**0.037**	η_p_^2^=0.127
Neutral-Distance	1.93 (1.04)	1.14 (0.21)	Group × task-instruction	F(1, 26)=1.04	0.318	η_p_^2^=0.038
			Image-type × task-instruction × group*	F(1.70, 44.2)=2.38	0.112	η_p_^2^=0.084

*Greenhouse-Geisser correction for non-sphericity.

†Huynh-Feldt correction for non-sphericity.

ANOVA, analysis of variance; df, degrees of freedom; FND, functional neurological disorder; HC, healthy controls; IQR, interquartile range; M, mean; Mdn, median; SD, standard deviation; η_p_^2^, partial eta squared.

Negative affect did not vary between groups at baseline or in the task. The only significant task-based main effect was image-type, reflecting significantly higher negative affect ratings following Negative relative to Positive (MD=0.56, p<0.001) and Neutral blocks (MD=0.59, p<0.001), in the complete sample. Ratings of negative affect did not differ between Positive and Neutral blocks (MD=0.03, p=1.000).

#### Dissociation

There were no significant group effects or interactions on depersonalisation ratings. There were also no group effects on derealisation ratings at baseline or in the task. However, the FND group reported significantly elevated amnesia compared with HCs at baseline and during the task.

There were significant main effects of task-instruction for derealisation and amnesia, with both elevated in the Distance compared with the Watch condition, across the sample. There was a significant interaction between image-type and task-instruction on amnesia ratings, spanning the full sample. Post hoc tests showed the effect of task-instruction was significant only for Neutral blocks, in which amnesia ratings were significantly higher in the Neutral-Distance condition compared with the Neutral-Watch condition (MD=0.20, p=0.016). There was no significant effect of task-instruction on amnesia ratings for Positive (MD=0.05, p=0.320) or Negative blocks (MD=0.06, p=0.193).

### Psychophysiological measures

#### Skin conductance (SC)

There was no significant effect of group at baseline or during the task, and no interactions between group and other factors (table 5).

The main effect of task instruction was significant, reflecting significantly higher SC in the Distance condition, relative to Watch (MD=0.54, p=0.023), across the full sample. The main effect of image-type was also significant in the complete sample, with SC values highest for Negative blocks, followed by Positive and Neutral blocks.

**Table 5:** Statistical values for psychophysiological variables

	FND (n=13)	HC (n=13)	Test/effect	Test statistics	p value	Effect size
**Skin conductance (microSiemens)**			**Independent samples t-test**			
Baseline: M (SD)	4.81 (1.96)	5.07 (1.25)	Group	t(24)=−0.41	0.343	g=0.156
Task: M (SD)			**ANOVA**			
Positive-Watch	4.5 (4.62)	2.93 (2.78)	Group	F(1, 24)=1.31	0.263	η_p_^2^=0.052
Positive-Distance	4.95 (5.28)	3.48 (3.37)	Image-type	F(2, 48)=3.85	**0.048**	η_p_^2^=0.119
Negative-Watch	5.5 (5.87)	3.4 (2.96)	Task-instruction	F(1, 24)=5.88	**0.023**	η_p_^2^=0.197
Negative-Distance	5.54 (5.85)	3.03 (2.78)	Image-type × group	F(2, 48)=2.05	0.14	η_p_^2^=0.079
Neutral-Watch	4.06 (3.86)	2.46 (2.05)	Image-type × task-instruction	F(2, 48)=4.19	**0.021**	η_p_^2^=0.149
Neutral-Distance	5.49 (5.84)	3.57 (3.64)	Group × task-instruction	F(1, 24)=0.23	0.634	η_p_^2^=0.010
			Image-type × task-instruction × group	F(2, 48)=0.15	0.861	η_p_^2^=0.006
**Heart rate (BPM)**			**Independent samples t-test**			
Baseline: M (SD)	80.6 (12.7)	72.5 (8.70)	Group	t(26)=1.95	**0.031**	g=0.714
Task: M (SD)			**ANOVA**			
Positive-Watch	0.27 (2.68)	−1.55 (2.66)	Group	F(1, 24)=5.46	**0.028**	η_p_^2^=0.185
Positive-Distance	0.52 (3.07)	−2.36 (2.54)	Image-type	F(2, 48)=12.1	**<0.001**	η_p_^2^=0.335
Negative-Watch	0.48 (3.82)	−2.82 (3.62)	Task-instruction	F(1, 24)=1.28	0.27	η_p_^2^=0.051
Negative-Distance	0.74 (3.65)	−1.82 (2.30)	Image-type × group	F(2, 48)=1.05	0.358	η_p_^2^=0.042
Neutral-Watch	1.82 (2.44)	−1.24 (2.28)	Image-type × task-instruction	F(2, 48)=1.99	0.148	η_p_^2^=0.077
Neutral-Distance	1.20 (2.92)	0.112 (2.88)	Group × task-instruction	F(1, 24)=1.67	0.208	η_p_^2^=0.065
			Image-type × task-instruction × group	F(2, 48)=5.23	**0.009**	η_p_^2^=0.179

ANOVA, analysis of variance; BPM, beats per minute; df, degrees of freedom; FND, functional neurological disorder; HC, healthy controls; M, mean; SD, standard deviation; η_p_^2^, partial eta squared.

There was a significant image-type × task-instruction interaction across all participants. The interaction was due to SC being significantly higher for Negative compared with Neutral blocks in the Watch condition (MD=1.19, p=0.013), but this difference was not significant in the Distance condition (MD=0.25, p=1.000).

Correlations between SC and FNS ratings were computed in the FND group only for the Negative-Watch, Negative-Distance and Neutral-Distance conditions, as these were associated with the highest FNS ratings (table 3). A positive correlation was observed between SC and FNS ratings in the Negative-Watch (r=0.628, p=0.011) and Neutral-Distance conditions (r=0.517, p=0.035), but not the Negative-Distance condition (r_s_=0.253, p=0.202).

Positive and negative affect ratings were not correlated with SC in either group in the Negative-Watch, Negative-Distance and Neutral-Distance conditions. Arousal ratings were not correlated with SC in either group in the Negative-Watch and Negative-Distance conditions. However, in the Neutral-Distance condition, SC was correlated inversely with momentary arousal ratings in FND (r_s_=−0.606, p=0.014), but not HCs (r_s_=−0.167, p=0.293).

#### Heart rate (HR)

HR was significantly elevated in the FND group compared with HCs during baseline and the task. Average HR accelerated during the task relative to baseline in the FND group, whereas it decelerated in HCs.

There was a significant main effect of image-type on HR during the task in the full sample, with HR highest for Neutral compared with both Positive (MD=1.25, p<0.001) and Negative blocks (MD=1.33, p=0.004). There was also a significant group × task-instruction × image-type interaction. In the Positive condition, the FND group had higher HR than HCs for Distance (MD=2.87, p=0.016) but not Watch blocks (MD=1.82, p=0.095). In the Negative condition, the FND group exhibited higher HR for both Watch (MD=3.30, p=0.033) and Distance blocks (MD=2.57, p=0.043). For Neutral images, the FND group displayed elevated HR compared with HCs in the Watch blocks (MD=3.05, p=0.003) but not in the Distance blocks (MD=1.09, p=0.347).

Correlations were carried out to assess relationships between HR and FNS ratings in the Negative-Watch, Negative-Distance and Neutral-Distance conditions (FND group only). In the FND group, HR was positively correlated with FNS ratings in the Negative-Watch (r=0.533, p=0.030) and Neutral-Distance conditions (r=0.672, p=0.006), but not in the Negative-Distance condition (r_s_=0.390, p=0.094).

In the Negative-Watch condition, HR was positively associated with negative affect ratings in the FND group (r_s_=0.526, p=0.032), but negatively associated with negative affect ratings in HCs (r_s_=−0.618, p=0.012), revealing a significant group difference in these coefficients (z=2.92, p=0.004).

HR was negatively correlated with negative affect ratings in the Negative-Distance condition in HCs (r_s_ *=*−0.566, p=0.022), but not in FND (r_s_=0.321, p=0.143). These coefficients also differed significantly (z=−2.18, p=0.030).

### Exploratory analyses

No significant relationships were observed between the key experimental outcomes and clinical variables or potential confounds (medication, mental health status).

## Discussion

We aimed to assess the feasibility of an experimental task designed to test the hypotheses that individuals with FND (motor symptoms/seizures) would display elevated autonomic reactivity and increased subjective FNS severity immediately following highly arousing affective stimulation.

### The influence of affective stimulation on subjective FNS

Subjective FNS were significantly elevated immediately after Negative compared with Positive and Neutral blocks. These results concur with two previous studies^31 32^ which showed altered sensorimotor function or subjective FNS in the context of affective processing tasks. However, the experimental design employed by Fiess *et al* 32 did not allow inferences to be made regarding which aspects of the task caused the changes, and Blakemore *et al* 31 measured sensorimotor functioning but not FNS or autonomic arousal.

Our findings provide novel evidence that negative affective events can cause a short-term increase in FNS severity, supporting the proposed role of emotional processing in the generation of FNS.^4 5^ These observations reflect the experiences of many individuals with FND who report that emotionally salient events can trigger or exacerbate their symptoms,^7 9^ although these processes may not be applicable in all cases.

### Autonomic reactivity

During the task, the effect of group and the interaction between group × image-type was not significant for SC. Previous findings on task-based SC have been variable across studies in FND samples, with elevated, reduced and comparable SC levels and/or phasic responses reported.^4 30^

There was a significant main effect of group on task-based HR, providing limited support for the hypothesis that the FND group would display enhanced autonomic reactivity. The overall HR deceleration observed in HCs was similar to that observed in other HC samples.^48 52^ In contrast, the FND group displayed overall HR acceleration, corresponding with previous reports.^23 53^

Positive correlations between SC/HR and FNS ratings in the Negative-Watch condition demonstrate a proximate relationship between autonomic arousal during affective stimulation and FNS severity. These findings are compatible with studies showing elevated objective and/or subjective peri-ictal autonomic arousal in FS,^10 27–29^ and those reporting associations between measures of autonomic arousal and neural network differences in paediatric FND samples.^26 54^ The results also accord with our previous observation of an association between phasic SC responses to negative affective images and self-reported ictal autonomic symptoms in FS.^12^ Together, these findings suggest a role for autonomic arousal as a triggering factor for FNS occurrence/aggravation.

### Intact subjective affect and arousal

The FND group did not differ to HCs in subjective affect and or perceived arousal at any timepoint, despite elevated HR and increased FNS severity ratings following affective stimulation in this FND group. There were also divergent relationships between HR and negative affect ratings in the FND and HC groups during the Negative-Watch condition.

Our results are relevant to models highlighting possible roles for altered interoception and bodily/emotional awareness in the pathophysiology of FND,^4 28 55^ suggesting possible differences in the way that bodily signals of affective arousal might be integrated with negative subjective emotional states in this population.

### The possible influence of voluntary cognitive detachment

In contrast to the Watch condition, subjective FNS severity did not differ between Negative and Neutral blocks in the Distance condition, with FNS ratings elevated to a similar degree in the Neutral-Distance and Negative-Distance conditions. Therefore, during exposure to both affectively neutral and negative events, the experience of cognitive detachment might contribute to the intensity of subjective FNS.

In the Neutral-Distance condition, there were significant positive correlations between SC/HR and FNS ratings, and an inverse relationship between SC and momentary arousal ratings in the FND group. These results indicate that during cognitive detachment in the context of neutral events, greater autonomic arousal was associated with increased FNS severity. Incongruously, this elevated autonomic activation was linked to *reduced* rather than increased *perceived arousal*.

There was a significant correlation between HR and negative affect ratings in HCs in the Negative-Distance condition that was not observed in the FND group. Cognitive detachment might therefore serve to reduce conscious awareness of physiological signals and modulate the experience of negative affect in those with FND.^17^

Regarding state dissociation, only dissociative amnesia was elevated in the FND group compared with HCs. These results conflict with elevated trait-depersonalisation scores in this sample, and contrast with previous studies reporting elevations in both detachment and compartmentalisation phenomena in FND.^33^ It is possible that some participants found the ‘Distance’ instruction challenging and may have used alternative strategies, such as emotional suppression.^56^

The main effect of task instruction on fatigue ratings across the full sample suggested that voluntary cognitive detachment resulted in elevated fatigue. It is possible that detachment is associated with increased cognitive load and may therefore be perceived as effortful. Future studies might seek to explore more closely the possible interactions between cognitive detachment and fatigue in clinical and non-clinical populations.^57^

### Consistently elevated pain and fatigue

Elevated pain and fatigue ratings in the FND group throughout the experiment support the possible relevance and burden of varied physical symptoms in individuals with FND.^36^ Interventions targeting pain, fatigue and other non-FNS somatic symptoms may be critical in FND management.

### Strengths and limitations

Our experimental design allowed us to examine short-term temporal relationships between affective stimulation and momentary FNS severity, alongside objective measures of affective reactivity, offering insights into the possible causal influences of affective stimulation and autonomic arousal in the pathogenesis of FNS. The experimental model of cognitive detachment is another strength. The influence of potential confounds was eliminated by recruiting HCs who were comparable to the FND group on most characteristics, and we excluded possible relationships between key dependent variables and medication/mental health status.

The study was limited by the small sample size, low statistical power, heterogeneous FND sample, omission of clinical controls and lack of objective FNS assessment. The inclusion of clinical controls is an important direction for future research, to better understand the specificity of these findings to FND. For example, comparison to physical health controls might reveal similar influences of affective stimulation and autonomic arousal on other physical symptoms. It will be important to also establish how the profile of results in FND differs to psychiatric controls, who might display alterations in psychological states (eg, negative affect) rather than changes in physical symptoms, during affective stimulation.

The FND sample here consisted predominantly of individuals with FMS as their primary symptom, and the findings may not generalise as well to individuals with FS, particularly those without FS warning symptoms who were excluded from this study.

Our experimental design did not allow us to examine the time-lagged influence of affective events on subjective FNS, which is reported by many individuals with FND. However, our broader project includes a remote monitoring study with ecological momentary assessment and wearable capture of autonomic variables. This remote monitoring study will provide us with further insights into the longer-term temporal dynamics between affectively significant events and FNS, in patients’ everyday lives.

FNS ratings may have been influenced in this study by demand characteristics, although this is unlikely because we did not observe group-specific alterations in ratings of other physical symptoms/states, the experimenter provided no information/suggestion about the hypotheses and the correlations between FNS ratings and autonomic variables indicate that the findings were not merely a result of top–down influences.

### Conclusions

This study provides novel evidence for a possible direct influence of negative affective stimulation on momentary subjective FNS, which was linked to changes in autonomic activation rather than altered subjective affect or perceived arousal. These findings help to unravel the complex influence of affective events on FNS and support models proposing roles for affective/autonomic mechanisms in FND. Interventions aimed at improving awareness, integration and regulation of autonomic signals might offer promise for adults with FND, as shown in children and young people with the diagnosis.^58 59^ Our future research will use a modified version of this task within a functional neuroimaging study, to examine the neural bases of these processes in larger samples with FS and FMS, in comparison to both healthy and psychiatric control groups.

## Data Availability

Data are available upon reasonable request.
