# Supplementary material for: Unravelling the influence of affective stimulation on functional neurological symptoms: a pilot experiment examining potential mechanisms
Source: J Neurol Neurosurg Psychiatry. 2023 Nov 14;95(5):461–70. doi: 10.1136/jnnp-2023-332364 (PMC11041613; doi:10.1136/jnnp-2023-332364)
Supplement: Supplementary data [file jnnp-2023-332364supp001.pdf]

Supplementary Materials

Supplementary Table 1. Eligibility criteria

	All participants	Functional neurological disorder (FND)	Healthy controls
**Inclusion criteria**	•18-65 years old.<br>•Normal or corrected eyesight.<br>•Fluency in English language.	•A primary diagnosis of FND with either motor symptoms OR seizures.*<br>*Participants with functional seizures were required to experience at least two seizures per month, with premonitory symptoms.	
**Exclusion criteria**	•Diagnosis of major cardiovascular disorder (e.g., heart disease).<br>•Diagnosis of major psychiatric disorder (e.g., psychosis, alcohol or substance dependence).<br>•Diagnosis of major neurological disorder (e.g., epilepsy, multiple sclerosis).	•Physical symptoms / disability impairing ability to perform tasks (e.g., severe/constant tremor, bilateral upper limb paralysis, seizure frequency > 10 per day).<br>•Medication that could significantly affect cardiovascular or cognitive functioning (e.g., beta-blockers, high-dose opiates).	•Current diagnosis of any major physical or mental health disorder.<br>•Lifetime diagnosis of functional neurological disorder.

Supplementary Table 2. Self-report questionnaire data

Questionnaire	Description	Scores by group		Comparison statistics
		FND (Total n=14)	HC (Total n=14)	
**Functional Neurological Symptoms Questionnaire**	Assesses the presence, frequency, severity and impact of FNS over the previous week. Scores are calculated for: total number of FNS, average severity (1-7), average impact (1-7), with higher scores indicating more symptoms/greater severity/impact.	See Table 2	-	-
**Patient Health Questionnaire – 15 (Kroenke et al., 2002): M (SD)**	Fifteen items assess the frequency of common somatic symptoms over the previous four weeks. Scores range from 0-30 – higher scores indicate more somatic symptoms.	13.1 (4.37)	3.0 (2.32)	t(19.8)=7.68, p<.001, g=2.82
**Patient Health Questionnaire – 9 (Kroenke et al., 2001): Mdn (IQR)**	Nine items measure the frequency of depressive symptoms over the past two weeks. Scores range from 0-27 – higher scores indicate elevated depressive symptoms.	11.5 (9.0)	1.5 (3.5)	U=12.5, z=-3.95, p<.001, r=.747
**Generalised Anxiety Disorder – 7 (Spitzer et al., 1999): Mdn (IQR)**	Seven items assess the frequency of generalised anxiety symptoms in the past two weeks. Scores range from 0-21 – higher scores indicate more anxiety.	7.5 (8.0)	1.5 (4.5)	U=31.0, z=-3.10, p=.001, r=.586
**Multiscale Dissociation Inventory (Briere, 2002): Mdn (IQR)**	A 30-item measure of the frequency of several forms of psychological dissociation over the preceding month. Raw scores are converted to T-scores (presented here). T-scores range from 0-170 – higher scores indicate greater dissociative symptomology.	**DENG**=60.0 (43.3)<br>**DEPR**=51.5 (48.3)<br>**DERL**=51.5 (30.3)<br>**ECON**=46.0 (10.5)<br>**MEMD**=55.0 (33.8)<br>**IDDIS**=47.0 (0.0)	**DENG**=50.0 (10.0)<br>**DEPR**=47.0 (0.0)<br>**DERL**=46.0 (0.0)<br>**ECON**=46.0 (4.0)<br>**MEMD**=48.5 (8.5)<br>**IDDIS**=47.0 (0.0)	**DENG**: U=57.5, z=-1.88, p=.062, r=.355<br>**DEPR**: U=49.0, z=-2.96, p=.024, r=.559<br>**DERL:** U=56.0, z=-2.33, p=.056, r=.44<br>**ECON:** U=92.5, z=-.296, p=.804, r=.056<br>**MEMD:** U=62.0, z=-1.72, p=.104, r=.325<br>**IDDIS:** U=84.0, z=-1.44, p=.541, r=.272
**Somatoform Dissociation Questionnaire – 20 (Nijenhuis et al., 1996):**	Twenty items examining the extent of various somatoform symptoms in the last year (e.g., sensory disturbances,	27.5 (9.25)	20.0 (0.0)	U=11.0, z=-4.22, p<.001, r=.798

**Mdn (IQR)**	speech/swallowing difficulties, pain). Scores range from 20-100 – higher scores indicate greater somatoform dissociation.			
**Toronto Alexithymia Scale – 20 (Bagby et al., 1994): M (SD)**	A 20-item measure of difficulties in emotional processing (i.e., identification/description of emotions, external cognitive orientation). Scores range from 20-100 – higher scores indicate greater alexithymia.	52.5 (11.0)	41.9 (10.8)	t(26)=2.59, p=.008, g=.949
**Traumatic Experiences Checklist (Nijenhuis et al., 2002): Mdn (IQR)**	A 33-item measure of lifetime traumatic experiences and their impact (e.g., bullying, life threatening illness, childhood abuse and neglect). Due to ethical concerns, we used a 29-item version, omitting the final four items which probe further details of abuse/maltreatment disclosures. Total scores ranged from 0-29 and impact scores for individual events ranged from 1-5. Higher scores signify greater trauma burden and impact.	**Total** =3.5 (5.3) **Impact=**12 (16.3)	**Total=**2.0 (3.5) **Impact=**8.0 (12.0)	**Total:** U=67.0, z=-1.44, p=.164, r=.272 **Impact:** U=67.5, z=-1.41, p=.164, r=.267

Notes. ECON=emotional constriction; DENG=disengagement; DEPR=depersonalisation; DERL=derealisation; FND=functional neurological disorder; FNS=functional neurological symptoms; IDDIS=identity dissociation; IQR=interquartile range; M=mean; Mdn=median; MEMD=memory disturbance; SD=standard deviation

Supplementary Table 3. Functional Neurological Symptoms Questionnaire

Please look at the symptoms in the table below and tell us whether you have experienced these functional neurological symptoms in the **past week.** If you mark yes to indicate that the symptom was present in the past week, please complete the additional columns to tell us **how frequent** the symptoms were, **how severe** (intense) they were, and **how much impact** they had on you.

When rating the average **severity** of symptoms, please choose a number from 1 to 7, where **1=Symptom not present; 2=Minimal; 3=Mild; 4=Moderate; 5=Moderately severe; 6=Severe; 7=Very severe**. When rating the **impact** of symptoms, please choose a number from 1 to 7, where **1=No impact at all; 2=Minimal impact; 3=Mild impact; 4=Moderate impact; 5=Moderately severe impact; 6=Severe impact; 7=Very severe impact**.

**FND Symptom**	**Present?** (circle or bold)	**Frequency** (circle or bold)	**Average severity (1-7)**	**Average impact (1-7)**
Weakness	Yes / No	Constant / daily / weekly / less than weekly		
Tremor	Yes / No	Constant / daily / weekly / less than weekly		
Dystonia (muscle spasms / fixed postures)	Yes / No	Constant (1) / daily (2) / weekly (3) / less than weekly (4)		
Walking / mobility difficulties	Yes / No	Constant / daily / weekly / less than weekly		
Myoclonus (muscle jerks)	Yes / No	Constant / daily / weekly / less than weekly		
Seizures*	Yes / No	Number of seizures in the last week:		
Numbness (loss of feeling)	Yes / No	Constant / daily / weekly / less than weekly		
Visual disturbances	Yes / No	Constant / daily / weekly / less than weekly		
Sensitivity to light/sound	Yes / No	Constant / daily / weekly / less than weekly		
Dizziness	Yes / No	Constant / daily / weekly / less than weekly		
Speech / swallowing difficulties	Yes / No	Constant / daily / weekly / less than weekly		
Cognitive difficulties (e.g., brain fog, memory lapses)	Yes / No	Constant / daily / weekly / less than weekly		
Other FND symptoms	Details:	Constant / daily / weekly / less than weekly		

Please tell us which FND symptom(s) is most severe and has the most impact on you.

*If you experience FND seizures, do you have warning symptoms? Yes / No

*If you experience warning symptoms before an FND seizure, what is the earliest or most consistent symptom(s) that you experience?

Supplementary Table 4. Standardised instructions – affective picture task

**Instructions 1**	In this task, you will be shown lots of different pictures on the screen, one at a time.<br>There will be 12 blocks of pictures, separated by brief breaks, when the word 'Rest' will be shown on screen.<br>During the breaks, please try to relax and stay focused on the screen.<br>Please press space to continue.
**Instructions 2**	Before some of the blocks of pictures, you will see the word 'Watch' on screen for a few seconds.<br>When you see this, please keep your eyes on the screen and just look at the subsequent pictures.<br>Please press space to continue.
**Instructions 3**	Before other blocks of pictures, you will see the word 'Distance' on screen.<br>When you see this, please try to minimise your reactions to the pictures by detaching yourself.<br>For example, you could imagine that you are an outside observer, distanced from your personal reactions to the pictures.<br>Please press space to continue.
**Instructions 4**	You will see a white fixation cross in the middle of the screen before every picture.<br>You will then see the word 'Watch' or 'Distance' to remind you whether to just look at the picture, or distance yourself from it.<br>You will then see the picture for a few seconds.<br>Some of the pictures may make you feel quite strong reactions; others may not affect you much at all.<br>Please keep your eyes focused on the screen throughout the task.<br>Please press space to continue.
**Instructions 5**	After every block of pictures, you will be asked several simple questions about how you feel, right then, in that moment.<br>First, you will be asked about your primary FND symptom, which was specified at enrolment to the study.*<br>You will be asked about different aspects of your current experiences and physical states.<br>You can choose your answers using the number keys on the keyboard.<br>Please try to answer as quickly and accurately as possible.<br>Press space to continue.
**Instructions 6**	You will now see some example pictures, with 'Watch' or 'Distance' instructions, so that you can practice the task.<br>Please press space to begin.
	**PRACTICE IMAGES x 6**
**Instructions 7**	You have now completed the practice items.<br>Please remember to look at the screen and stay as still as possible during this task.<br>Remember, when you see the word 'Watch', just look at the pictures.<br>When you see the word 'Distance', try to minimise your reactions by detaching yourself.<br>Feel free to ask any questions now.[#]<br>Please press space to begin.

*Sentence omitted from healthy control version
[#]Experimenter answered questions in full and ensured the task was understood fully prior to participants commencing

Supplementary Table 5. IAPS images

Image	Description	Category	Mean Arousal	SD Arousal	Mean Valence	SD Valence
1300	PitBull	NHA	6.79	1.84	3.55	1.78
7380	RoachPizza	NHA	5.88	2.44	2.46	1.42
2811	Gun	NHA	6.9	2.22	2.17	1.38
3064	Mutilation	NHA	6.41	2.62	1.45	0.97
9921	Fire	NHA	6.52	1.94	2.04	1.47
3030	Mutilation	NHA	6.76	2.1	1.91	1.56
9622	Jet	NHA	6.26	1.98	3.1	1.9
2683	War	NHA	6.21	2.15	2.62	1.78
2730	NativeBoy	NHA	6.8	2.21	2.45	2.25
6530	Attack	NHA	6.18	2.02	2.76	1.86
1525	Attack dog	NHA	6.51	2.25	3.09	1.72
2661	Baby	NHA	5.76	2.13	3.9	2.49
1050	Snake	NHA	6.87	1.68	3.46	2.15
3022	Scream	NHA	5.88	2.08	3.7	1.91
3400	Severed Hand	NHA	6.91	2.22	2.35	1.9
6022	Assault	NHA	6.09	2.47	2.14	1.55
9910	CarAccident	NHA	6.2	2.16	2.06	1.26
3102	BurnVictim	NHA	6.58	2.69	1.4	1.14
6415	DeadTiger	NHA	6.2	2.31	2.21	1.51
9620	Shipwreck	NHA	6.11	2.1	2.7	1.64
6312	Abduction	NHA	6.37	2.3	2.48	1.52
1201	Spider	NHA	6.36	2.11	3.55	1.88
2981	DeerHead	NHA	5.97	2.12	2.76	1.94
6212	Soldier	NHA	6.01	2.44	2.19	1.49
9300	Dirty	NHA	6	2.41	2.26	1.76
9500	Porpoises	NHA	5.82	2.29	2.42	1.73
9254	Assault	NHA	6.04	2.35	2.03	1.35
9405	SlicedHand	NHA	6.08	2.4	1.83	1.17
9902	CarAccident	NHA	6	2.15	2.33	1.38
6370	Attack	NHA	6.44	2.19	2.7	1.52
1932	Shark	NHA	6.47	2.2	3.85	2.11
3100	BurnVictim	NHA	6.49	2.23	1.6	1.07
1301	Dog	NHA	5.77	2.18	3.7	1.66
9252	DeadBody	NHA	6.64	2.33	1.98	1.59
3250	OpenChest	NHA	6.29	1.63	3.78	1.72
3150	Mutilation	NHA	6.55	2.2	2.26	1.57
9810	KKKRally	NHA	6.62	2.26	2.09	1.78

6570	Suicide	NHA	6.24	2.16	2.19	1.72
6315	BeatenFem	NHA	6.38	2.39	2.31	1.69
3063	Mutilation	NHA	6.35	2.6	1.49	0.96
8178	Cliffdiver	PHA	6.82	2.33	6.5	2
7270	Icecream	PHA	5.76	2.21	7.53	1.73
8080	Sailing	PHA	6.65	2.2	7.73	1.34
4689	EroticCouple	PHA	6.21	1.74	6.9	1.55
8341	WingWalker	PHA	6.4	2.27	6.25	1.86
4311	EroticFemale	PHA	6.67	2.19	6.66	1.76
8034	Skier	PHA	6.3	2.16	7.06	1.53
8370	Rafting	PHA	6.73	2.24	7.77	1.29
8186	SkySurfer	PHA	6.84	2.01	7.01	1.57
4677	EroticCouple	PHA	6.19	2.08	6.58	1.65
1650	Jaguar	PHA	6.23	1.99	6.65	2.25
2216	Children	PHA	5.83	2.2	6.41	1.9
8179	Bungee	PHA	6.99	2.35	6.48	2.18
4653	EroticCouple	PHA	5.83	2.07	6.56	1.65
8501	Money	PHA	6.44	2.29	7.91	1.66
8470	Gymnast	PHA	6.14	2.19	7.74	1.53
5621	Skydivers	PHA	6.99	1.95	7.57	1.42
4608	EroticCouple	PHA	6.47	1.96	7.07	1.66
5470	Astronaut	PHA	6.02	2.26	7.35	1.62
8170	Sailboat	PHA	6.12	2.3	7.63	1.34
4695	EroticCouple	PHA	6.61	1.88	6.84	1.53
5626	Hanglider	PHA	6.1	2.19	6.71	2.06
8499	Rollercoaster	PHA	6.07	2.31	7.63	1.41
4664	EroticCouple	PHA	6.72	2.08	6.61	2.23
8190	Skier	PHA	6.28	2.57	8.1	1.39
5450	Liftoff	PHA	5.84	2.4	7.01	1.6
8496	Waterslide	PHA	5.79	2.26	7.58	1.63
7230	Turkey	PHA	5.52	2.32	7.38	1.65
8200	Waterskier	PHA	6.35	1.98	7.54	1.37
4607	EroticCouple	PHA	6.34	2.16	7.03	1.84
8400	Rafters	PHA	6.61	1.86	7.09	1.52
8490	Rollercoaster	PHA	6.68	1.97	7.2	2.35
7502	Castle	PHA	5.91	2.31	7.75	1.4
5629	Hiker	PHA	6.55	2.11	7.03	1.55
4611	EroticCouple	PHA	6.04	2.11	6.62	1.82
8300	Pilot	PHA	6.14	2.21	7.02	1.6
4652	EroticCouple	PHA	6.62	2.04	6.79	2.02
8191	IceClimber	PHA	6.19	2.17	6.07	1.73

8180	CliffDivers	PHA	6.59	2.12	7.12	1.88
4643	EroticCouple	PHA	6.01	2	6.84	1.54
5531	Mushroom	Neutral	3.69	2.11	5.15	1.45
7009	Mug	Neutral	3.01	1.97	4.93	1
7037	Trains	Neutral	3.71	2.08	4.81	1.12
2396	Couple	Neutral	3.34	1.83	4.91	1.05
2880	Shadow	Neutral	2.96	1.94	5.18	1.44
2516	Elderly woman	Neutral	3.5	1.88	4.9	1.43
2493	NeutralMale	Neutral	3.34	2.1	4.82	1.27
2890	Twins	Neutral	2.95	1.87	4.95	1.09
7207	Beads	Neutral	3.57	2.25	5.15	1.46
7217	ClothesRack	Neutral	2.55	1.65	5	0.78
5510	Mushroom	Neutral	2.82	2.18	5.15	1.43
2102	NeuMan	Neutral	3.03	1.87	5.16	0.96
7059	KeyRing	Neutral	2.73	1.88	4.93	0.81
2514	Woman	Neutral	3.5	1.81	5.19	1.09
7000	RollingPin	Neutral	2.42	1.79	5	0.84
2393	FactoryWorker	Neutral	2.93	1.88	4.87	1.06
7036	Shipyard	Neutral	3.32	2.04	4.88	1.08
2595	Women	Neutral	3.71	1.88	4.88	1.24
7010	Basket	Neutral	1.55	1.36	4.95	1.43
7491	Building	Neutral	2.6	1.95	4.87	0.94
2840	Chess	Neutral	2.43	1.82	4.91	1.52
7235	Chair	Neutral	2.68	1.9	4.85	1.13
7038	Shoes	Neutral	3.01	1.96	4.82	1.2
7055	LightBulb	Neutral	3.02	1.83	4.9	0.64
7160	Fabric	Neutral	3.07	2.07	5.02	1.1
5534	Mushrooms	Neutral	3.14	2.03	4.84	1.44
2385	Girl	Neutral	3.64	1.81	5.2	1.32
7034	Hammer	Neutral	3.06	1.95	4.95	0.87
7950	Tissue	Neutral	2.28	1.81	4.94	1.21
7187	AbstractArt	Neutral	2.3	1.75	5.07	1.02
7179	Rug	Neutral	2.88	1.97	5.06	1.05
9070	Boy	Neutral	3.63	2.03	5.01	1.89
7041	Baskets	Neutral	2.6	1.78	4.99	1.12
2512	Man	Neutral	3.46	1.75	4.86	0.84
5532	Mushrooms	Neutral	3.79	2.2	5.19	1.69
7006	Bowl	Neutral	2.33	1.67	4.88	0.99
7090	Book	Neutral	2.61	2.03	5.19	1.46
6150	Outlet	Neutral	3.22	2.02	5.08	1.17

2038	NeuWoman	Neutral	2.94	1.93	5.09	1.35
7002	Towel	Neutral	3.16	2	4.97	0.97

Notes. NHA=negative high arousal; PHA=positive high arousal; SD=standard deviation
